# Y-Chromosome Variation in Hominids: Intraspecific Variation Is Limited to the Polygamous Chimpanzee

**DOI:** 10.1371/journal.pone.0029311

**Published:** 2011-12-27

**Authors:** Gabriele Greve, Evguenia Alechine, Juan J. Pasantes, Christine Hodler, Wolfram Rietschel, Terence J. Robinson, Werner Schempp

**Affiliations:** 1 Institute of Human Genetics, University of Freiburg, Freiburg, Germany; 2 Servicio de Huellas Digitales Genéticas, School of Pharmacy and Biochemistry, University of Buenos Aires, Buenos Aires, Argentina; 3 Department of Biochemistry, Genetics and Immunology, University of Vigo, Vigo, Spain; 4 Wilhelma der Zoologisch Botanische Garten, Stuttgart, Germany; 5 Evolutionary Genomics Group, Department of Botany and Zoology, University of Stellenbosch, Stellenbosch, South Africa; University of Florence, Italy

## Abstract

**Background:**

We have previously demonstrated that the Y-specific ampliconic fertility genes *DAZ* (deleted in azoospermia) and *CDY* (chromodomain protein Y) varied with respect to copy number and position among chimpanzees (*Pan troglodytes*). In comparison, seven Y-chromosomal lineages of the bonobo (*Pan paniscus*), the chimpanzee's closest living relative, showed no variation. We extend our earlier comparative investigation to include an analysis of the intraspecific variation of these genes in gorillas (*Gorilla gorilla*) and orangutans (*Pongo pygmaeus*), and examine the resulting patterns in the light of the species' markedly different social and mating behaviors.

**Methodology/Principal Findings:**

Fluorescence *in situ* hybridization analysis (FISH) of *DAZ* and *CDY* in 12 Y-chromosomal lineages of western lowland gorilla (*G. gorilla gorilla*) and a single lineage of the eastern lowland gorilla (*G. beringei graueri*) showed no variation among lineages. Similar findings were noted for the 10 Y-chromosomal lineages examined in the Bornean orangutan (*Pongo pygmaeus*), and 11 Y-chromosomal lineages of the Sumatran orangutan (*P. abelii*). We validated the contrasting *DAZ* and *CDY* patterns using quantitative real-time polymerase chain reaction (qPCR) in chimpanzee and bonobo.

**Conclusion/Significance:**

High intraspecific variation in copy number and position of the *DAZ* and *CDY* genes is seen only in the chimpanzee. We hypothesize that this is best explained by sperm competition that results in the variant *DAZ* and *CDY* haplotypes detected in this species. In contrast, bonobos, gorillas and orangutans—species that are not subject to sperm competition—showed no intraspecific variation in *DAZ* and *CDY* suggesting that monoandry in gorillas, and preferential female mate choice in bonobos and orangutans, probably permitted the fixation of a single Y variant in each taxon. These data support the notion that the evolutionary history of a primate Y chromosome is not simply encrypted in its DNA sequences, but is also shaped by the social and behavioral circumstances under which the specific species has evolved.

## Introduction

The human and chimpanzee male-specific regions of the Y chromosome (MSY) are fully sequenced [1]–[4]. These meiotically non-recombining regions harbor extensive palindromes that include ampliconic gene families. Most of these genes are known to play an important role in male fertility [1], and are thus under significant selective pressure. Although sequence data are not available for the bonobo, gorilla and orangutan MSY regions, molecular investigations [5], including molecular cytogenetic studies [6]–[10], detect similar palindromic repeats within MSY of these species.

We have previously identified variation in the copy number and positions of the fertility genes *DAZ* and *CDY* in the chimpanzee (*P. troglodytes*) [11]. Interestingly, and in marked contrast to chimpanzees, bonobo specimens (*P. paniscus*) were invariant. We hypothesized that this contrast reflected differences in social and mating behavior evident among the species [11]. Here we extend our original findings by examining *DAZ* and *CDY* intraspecific variation in other great apes, specifically within *Gorilla* and *Pongo*.

Gorillas are divided into two species, *G. gorilla* and *G. beringei*, each comprising two subspecies [12], [13]. The first of these, the western lowland gorilla (*G. g. gorilla*) is found in the moist tropical forests of western and central Africa. The so-called “cross-river” gorilla (*G. g. diehli*) survives in a small area on the border of Nigeria and Cameroon. At least 1000 km separates these taxa from the eastern gorilla (*G. beringei*). The eastern lowland gorilla (*G. b. graueri*) occurs in moist tropical forests in the east of the Democratic Republic of Congo (DRC), while the nominate subspecies (*G. b. beringei*) extends into the cloud forests of the Virunga Volcanic Massif and the Bwindi Impenetrable National Park, an area bordered by the DRC, Uganda and Ruanda. The orangutans are similarly divided into two species, the Sumatran orangutan (*Pongo abelii*) and the Bornean orangutan (*P. pygmaeus*) [14]–[17]. The former is restricted to the north-western parts of Sumatra [13]. *Pongo pygmaeus*, on the other hand, includes *P. p. pygmaeus* (north-western Borneo on the border of Kalimantan and Sawarak), *P. p. wurmbii* (central Kalimantan), and *P. p. morio* (north-eastern Borneo, specifically in east Kalimantan and Sabah) [18].

The Y chromosome comprises 2–3% of the haploid genome in both human and gorilla—species distinguished by the largest of the simian Y chromosomes. Both taxa are characterized by significant amounts of constitutive heterochromatin on the long arm i.e., Yq [7]. It should be noted that most (and probably all) published cytogenetic studies (banding and FISH) of gorilla Y chromosomes are of the western lowland gorilla [6,19 and references therein, 20–24]. The constitutive heterochromatin on Yq in the western lowland gorilla is subdivided into three blocks separated by euchromatic segments. FISH-hybridization has shown that the fertility genes *DAZ* and *CDY* co-hybridize within the proximal euchromatic segment that is delimited by adjacent heterochromatic blocks on the gorilla Y chromosome; *CDY* signals were also detected in the pericentromeric region of this chromosome [7], [9].

The Y chromosomes of the two orangutan species are somewhat smaller than those of human and gorilla. Interestingly they differ—a pericentric inversion distinguishes the metacentric *P. pygmaeus* element from its *P. abelii* submetacentric ortholog. This inversion resulted in the transfer of part of the *DAZ/CDY*-cluster to the proximal short arm (Yp) of the Y chromosome [7], [9]. The two species are also distinguished by the presence of a Y-satellite (Yqs), and a nucleolus organizing region (NOR) on distal Yq, both of which are specific to the Sumatran orangutan, *P. abelii* [25].

Here we provide new insights to the variation of ampliconic fertility genes *DAZ* and *CDY* in apes. We report on the structural arrangement of these genes in the gorilla and orangutan, and confirm the intraspecific variation of *DAZ* and *CDY* in the common chimpanzee and their homogeneity in bonobo by gene dosage analysis using qPCR. This approach allowed us to assess correspondence between the FISH results and the underlying genomic sequences. Both genes occur in the male-specific region of the Y chromosome and are expressed exclusively in the testis of human [1] and chimpanzee [4] making their location, and function, ideal for exploring the association between chromosomal restructuring and differences in the social and mating behavior of these species.

## Results

### (i) Comparative mapping of *DAZ* and *CDY*

**Figure 1:**
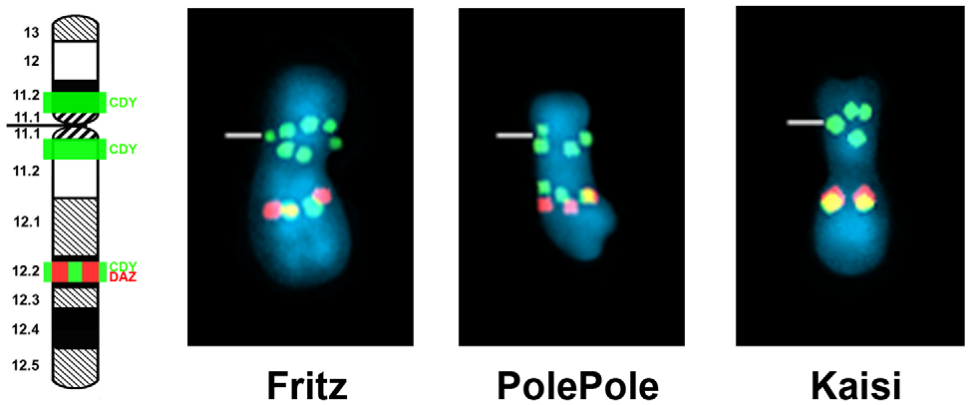
FISH mapping of ampliconic genes *DAZ* and *CDY* on Y chromosomes of gorilla. FISH patterns for *DAZ* (red) and *CDY* (green) illustrated for the western lowland gorillas “Fritz” and “PolePole”, and eastern lowland gorilla “Kaisi”. The band locations of each gene are shown on the ideograms to the left. Yellow signals result from overlapping of red and green signals. Centromeres are marked by horizontal lines. Band nomenclature follows the ISCN [71].
- **Pongo.** the outcome of FISH experiments on the Y chromosomes of 13 specimens of *P. pygmaeus* (the Bornean orangutan), of which 10 were wild-born, and 17 specimens of *P. abelii* (the Sumatran orangutan), of which 11 were wild-born, are presented in Tables S2 and S3. *DAZ* and *CDY* co-hybridize as a strong signal cluster on Yq13 in the Bornean orangutan (Figure 2). No obvious variation was noted among the 13 specimens investigated. The FISH patterns for the Sumatran orangutan NOR-bearing satellited Y (i.e., Yqs) clearly show two variants (Figure 3). Yqs-type 1 exhibits a pattern similar to that of the Bornean animals (hybridization only at Yq13). This was found in 11 specimens (eight of which are wild-born) (Table S3). Yqs-type 2 shows additional signals for *DAZ* and *CDY* on the short arm of the Y, specifically at Yp13.1, and this was detected in six specimens of *P. abelii* (three of which wild-born) (Table S3).

Y chromosomal variation was determined among gorilla and orangutan specimens by FISH using human-derived DNA probes specific for *DAZ* and *CDY*. Hybridization signals were scored on metaphase and prometaphase Y chromosomes of *Gorilla* (N = 21) and *Pongo* (N = 30), and the outcomes presented in detail below:

- **Gorilla.** Comparative FISH data for 20 specimens of western lowland gorilla (*G. g. gorilla*) representing 12 wild-born males and one captive specimen of eastern lowland gorilla (*G. b. graueri*) are presented in Table S1. Our data are consistent with earlier reports [7], [9] that show *DAZ* and *CDY* to localize at Yq12.2, a euchromatic segment embedded between two heterochromatic blocks on the long arm of the gorilla Y chromosome. Two additional pericentromeric *CDY* signals were evident in all specimens examined of this species (Figure 1).

**Figure 2:**
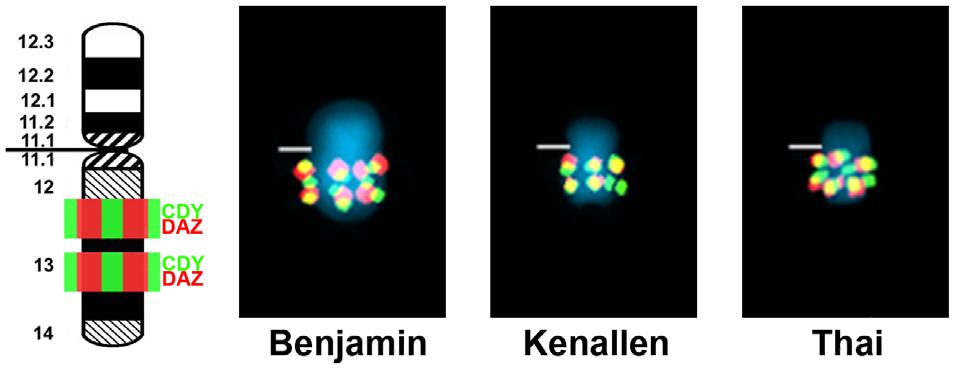
FISH mapping of ampliconic genes *DAZ* and *CDY* on Y chromosomes of Bornean orangutan. FISH patterns for *DAZ* (red) and *CDY* (green) illustrated for the Bornean orangutans “Benjamin”, “Thai” and “Kenallen”. The band locations of each gene are shown on the ideograms to the left. Yellow signals result from overlapping of red and green signals. Centromeres are marked by horizontal lines. Band nomenclature follows the ISCN [71].

**Figure 3:**
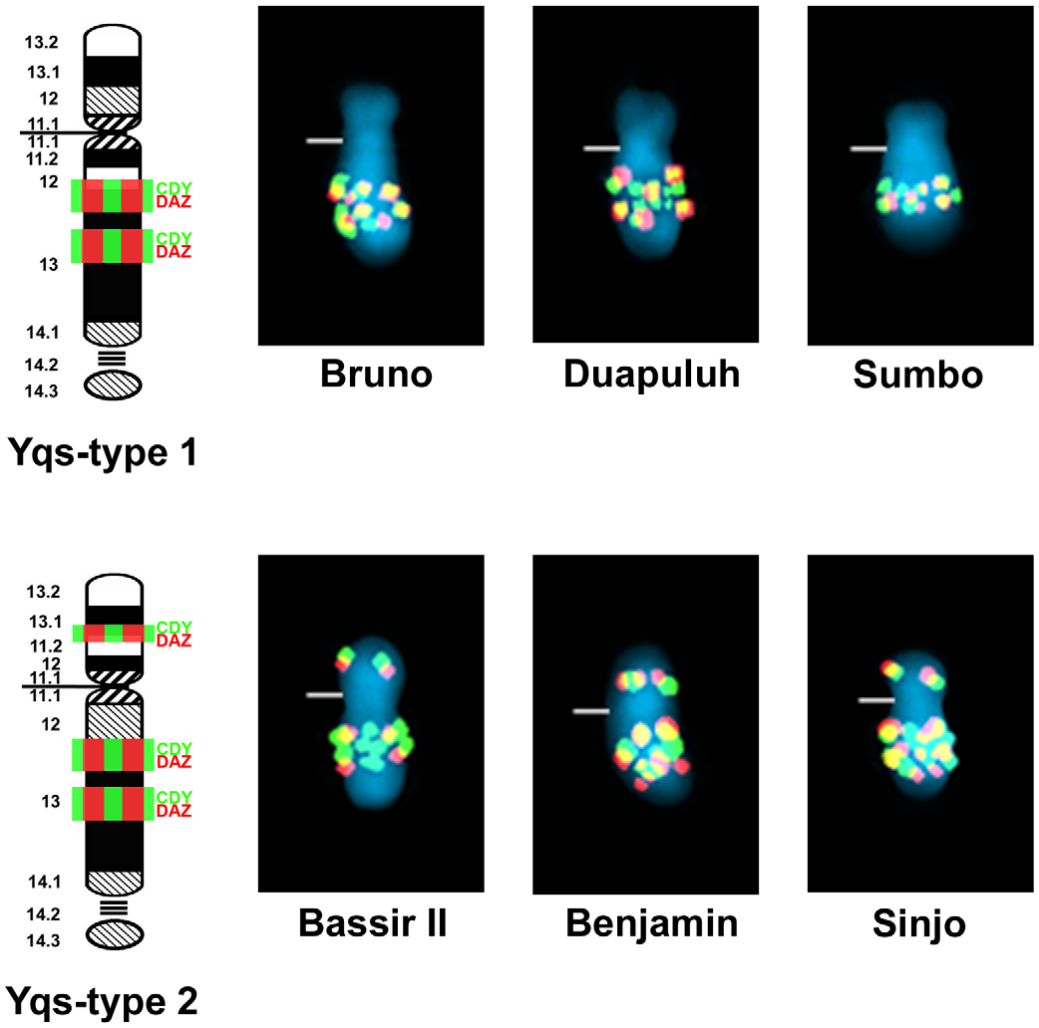
FISH mapping of ampliconic genes *DAZ* and *CDY* on Y chromosomes of Sumatran orangutan. FISH patterns for *DAZ* (red) and *CDY* (green) illustrated for Sumatran orangutans “Bruno”, “Duapuluh” and “Sumba” showing Yqs-type 1, as well as “Bassir II”, “Sinjo” and “Benjamin” showing Yqs-type 2. The band locations of each gene are shown on the ideogram to the left. Yellow signals result from overlapping of red and green signals. Centromeres are marked by horizontal lines. Band nomenclature follows the ISCN [71].

### (ii) Detection of intraspecific copy number variation of *DAZ* and *CDY* by qPCR

Our previous work showed that 10 of 11 Y-chromosomal lineages in *P. troglodytes* [11] varied with respect to the number and arrangement of the *DAZ* and *CDY* FISH-signals; no variation was evident among seven Y-chromosomal lineages of the bonobo, *P. paniscus*. We re-examine these findings using quantitative real-time PCR, considered to offer an independent and fine-scale analysis of copy number variation of *DAZ* and *CDY* in these species. The ratio *DAZ/SRY* and *CDY/SRY* was determined from genomic DNA of 10 specimens of the common chimpanzee (Table S4), and 10 specimens of the bonobo (Table S5) following standardization of template DNA (quantity and quality) in each reaction. Student's *t*-test showed no significant differences between the four replicates performed for each sample (p>0.05). Although genomic DNA of the chimpanzee specimens “Clint”, “Adam”, “Hans” and “Sixtus”, and the bonobo specimen “Clyde” from our former study [11] were no longer available, it seems reasonable, based on the correspondence between the quantification of *DAZ* and *CDY* copy number by qPCR and Y-chromosomal FISH-results for specimens with complementary data sets, that this similarly holds in all cases. The finding of two copies of *DAZ* and *CDY* in all 10 bonobos is consistent with a single FISH-signal for both genes [11] indicating that the bonobo is invariant with respect to location and copy number of these genes (Table S6). The common chimpanzee, on the other hand, shows either two or four *DAZ* copies that correspond to one or two FISH-signals on the Y. This is similarly reflected by *CDY* which varies between two and six copies among specimens, in agreement with the number of FISH-signals detected on the *P. troglodytes* Y chromosomes (Table S7).

No consistent results were obtained from genomic DNA of gorilla and orangutan specimens by qPCR using conditions that were identical to those used for amplification from chimpanzee and bonobo genomic DNA. Unfortunately Y-chromosomal sequences for *DAZ* and *CDY* are not available for either species (only female genomes are accessible http://www.sanger.ac.uk/resources/downloads/gorilla/; [26]) thus hampering experimental trouble shooting and precluding an assessment of correspondence between the FISH results and the genomic sequences in these taxa.

## Discussion

The Y chromosome differs from the X and from all autosomes with respect to both its bipartite structure and its function. Although sharing a pseudoautosomal region (PAR) with the X (this permits recombination between the sex chromosomes ensuring proper meiotic segregation), the independent male-specific region of the Y (MSY) can accumulate mutations that may lead to disturbances in sex determination [27], [28], and fertility [29]. Rare variants, and rearrangements of the MSY by inversions and/or the presence of satellited Y chromosomes, have been documented in humans [30]–[35], chimpanzees and bonobos [36].

We have previously argued [11] that copy number variation and the arrangement of the fertility genes *DAZ* and *CDY* among chimpanzees and their invariant presence in bonobo, the chimpanzee's closest living relative, had a behavioral/social foundation. We suggested that multiple chimpanzee males copulate with a receptive female during a short period of visible anogenital swelling, and this may place significant selection on fertility genes through sperm competition. In bonobos, however, female mate choice may make sperm competition redundant. Ovulation is concealed by prolonged anogenital swelling in this species, and because female bonobos can occupy high-ranking positions in the group, they are able to express mate choice more freely. Here we both confirm these results and extend them to orangutan and gorilla. Additionally we validate our earlier findings on the chimpanzee and bonobo by testing for gene dosage differences using real-time qPCR.

Our comparative FISH-studies (Fig. 1–3) showed no variation in copy number or location of *DAZ* and *CDY* in twelve Y-chromosomal lineages of the western lowland gorilla (*G. g. gorilla*), and in the Y-chromosomal lineage of the eastern lowland gorilla (*G. b. graueri*). This would suggest that the species is invariant—although this must be tempered by the absence of similar data for the cross-river gorilla, *G. g. diehli*, and the mountain gorilla, *G. b. beringei*. Similarly, the lack of variation among 10 Y-chromosomal lineages of the Bornean orangutan (*Pongo pygmaeus*) suggests that this too is monomorphic at these loci. In contrast, the Sumatran orangutan (*P. abelii*) differs from its conspecific by the presence of an NOR-bearing satellited Y chromosome (Yqs) [25], and by showing two Yqs lineages based on the *DAZ* and *CDY* FISH patterns. The presence of additional signals at Yp in three of 11 Y-chromosomal lineages may be explained by a pericentric inversion of the Y chromosome within the male population of *P. abelii*, perhaps reflecting a founder event in this taxon. Interestingly, pericentric inversions and rearrangements of genes within the non-recombining part of the Y chromosome are tolerated in humans, and most likely have no impact on fertility [35], suggesting that the different Y-lineages detected in *P. abelii* are naturally occurring variants in the wild.

Should this interpretation hold, i.e., that the number and location of the *DAZ* and *CDY* fertility genes are essentially invariant in gorillas and orangutans, it begs the question whether there is evidence to suggest that, as with the chimpanzee and bonobo, this too reflects social and mating behavioral differences. Both *Gorilla* and *Pongo* show monoandrous mating behavior, a high degree of sexual dimorphism, and a hierarchy that is governed by a dominant male [18], [37]. Typically, western lowland gorillas (*G. g. gorilla*) live in social groups composed of a single dominant male (silverback) and several adult females with their offspring; the silverback monopolizes breeding [38]–[41]. Social groups of mountain gorillas (*G. b. beringei*) contain either a single, or multiple silverbacks [42]. However, even in multimale groups a dominant male sires the majority of offspring, although this may be ameliorated by female mate choice [43], [44].

It is rather more difficult to categorize the mating systems of the Sumatran (*P. abelii*) and the Bornean (*P. pygmaeus*) orangutans because of their semi-solitary lifestyles, large home ranges, and the paucity of life history data for these species [45,46, reviewed in 18,47]. Orangutans seem to form a network of loosely associated individual-based fission-fusion societies, established around a dominant male [46], [48]. Dominant males and females normally mate within the context of a consortship that is often initiated by females at the time of presumed fecundity. It appears that females express mating preferences (for and against certain males), either by initiating consortship, or by resisting mating attempts [49], [50]. Females may also initiate consortships with low-ranking males, mainly during periods of unstable male ranks [51, reviewed in 18,47].

Given these findings it is reasonable to ask whether additional chromosomal data and information from other parts of the genome detect similar contrasting patterns of variability to those illustrated by *DAZ* and *CDY*. There are several comparative population genetic studies on human and great ape species based on autosomal, gonosomal and mitochondrial DNA variation that permit estimates of variation within and among hominid species [52]–[56]. These data suggest that humans generally have lower levels of genetic variation than do the great ape species, a pattern that can be attributed to a recent founder effect resulting in a low effective population size for humans [57], [58]. Additionally, fine-scale analyses of other homonid species have been conducted. For example, a study of five autosomal loci among African great apes showed the common chimpanzees to have the highest nucleotide diversity, with bonobos and gorillas possessing somewhat less variation [59]. Similarly, X-chromosome sequence variation in *Homo* and *Pan* indicated that chimpanzees have twice the diversity found in humans, whereas bonobos have even less diversity than humans [60]. In a further study Yu and co-workers [61] compared the sequence of 50 DNA segments randomly chosen from the non-coding, non-repetitive parts of the human genome to those of bonobos, chimpanzees and western lowland gorillas. Summed across these 50 segments, the highest nucleotide diversity was found in chimpanzees and gorillas, while bonobos carry the lowest nucleotide diversity–lower than in humans. Although these investigations mostly reflect differences in population histories among species [58], the presence of consistent *DAZ*/*CDY* patterns in species showing high genetic diversity would tend to support the notion that low levels of sperm competition are associated with low intraspecific Y-chromosomal variation.

In conclusion, the major findings of this study are: (i) That the contrasting patterns of *DAZ* and *CDY* variability in chimpanzees (*P. troglodytes*) and bonobos (*P. paniscus*), initially suggested by comparative FISH [11], are similarly reflected by real-time qPCR data. (ii) Although chimpanzee and bonobo share promiscuous mating behaviors, it is only in chimpanzees that male dominance is sufficiently developed to influence sperm competition [62], [63]. This results in high selective pressure on male fertility genes. Bonobos on the other hand, are characterized by a matriarch-dominated societal structure [63,64, reviewed in 65] which, coupled to concealed ovulation [66], permits female mate choice, thus rendering sperm competition redundant. (iii) That monoandrous mating in gorillas (*G. gorilla*) [37]–[41] and female mate choice in orangutans (*P. pygmaeus* and *P. abelii*) [18], [49], [50] similarly accounts for the dearth of intraspecific Y-chromosomal variation in the ampliconic fertility genes *DAZ* and *CDY* among Y-chromosomal lineages in these species.

## Materials and Methods

### Ethics Statement

The non-human primate blood samples were obtained serendipitously by zoo-physicians during anesthesia of animals for clinical conditions. Protocols followed the guidelines of the relevant Ethic Committees (Zoo Antwerpen, Apenheul Primate Park Apeldoorn, Zoo Basel, Zoo Berlin, Zoo Cologne, Zoo Duisburg, Zoo Frankfurt, Zoo Karlsruhe, Zoo Krefeld, Zoo Leipzig, Tierpark Hellabrunn München, Zoo Nürnberg, SanDiego Zoo, Zoo Seeteufel Studen CH, Zoo Wilhelma Stuttgart, Zoo Wuppertal, Yerkes National Primate Research Center, Zoo Zürich) for research involving non-human primates.

### Blood samples

Peripheral blood samples (heparinized for chromosome preparation and EDTA for DNA preparation) were obtained from animals housed in the following facilities: Zoo Antwerpen, Apenheul Primate Park Apeldoorn, Zoo Basel, Zoo Berlin, Zoo Cologne, Zoo Duisburg, Zoo Frankfurt, Zoo Karlsruhe, Zoo Krefeld, Zoo Leipzig, Tierpark Hellabrunn München, Zoo Nürnberg, San Diego Zoo, Zoo Seeteufel Studen CH, Zoo Wilhelma Stuttgart, Zoo Wuppertal, Yerkes National Primate Research Center, Zoo Zürich. Details on the origin and status of the gorilla and orangutan specimens are presented in Tables S1, S2 and S3.

### Chromosomal preparations

Preparations were made directly from peripheral blood lymphocytes according to standard methods with minor modifications [67]. Mitotic spreads were dehydrated in an ethanol series (70%, 90% and 100% each for 3 min), then air dried and stored at −80°C. Prior to *in situ* hybridization, the slides were again passed through an alcohol series (70%, 90% and 100% each for 3 min) and then air dried.

### FISH analysis

All FISH-assays were performed on metaphase and prometaphase spreads following Schempp et al. [6]. Slides were treated with RNase and pepsin as described [68]. Chromosome *in situ* suppression (CISS) was applied to gene clones cos6B7 and cos7F11 for *DAZ* [69], and cos2A49 for *CDY* [34]. For two-color detection, double-hybridization experiments were performed with biotinylated and digoxigenin (DIG)-labeled probes. Biotinylated probes were detected with FITC-conjugated avidin (Vector Laboratories) and DIG-labeled probes using anti-DIG-mouse antibodies (Sigma) followed by TRITC-conjugated goat anti-mouse antibodies (Sigma). After FISH the slides were counterstained with DAPI (4′,6-diamidino-2-phenylindole; 0.14 µg/ml) and mounted in Vectashield (Vector Laboratories). Preparations were evaluated using a Zeiss Axiophot epifluorescence microscope equipped with single-bandpass filters for excitation of red, green, and blue (Chroma Technologies). During exposures, only excitation filters were changed allowing for pixel-shift-free image recording. Metaphases were photographed with a cooled camera coupled to the microscope. The DAPI, FITC and TRITC images were merged using the image software Adobe Photoshop.

### Quantitative real-time PCR (qPCR)

qPCR was carried out using two primer pairs for each *DAZ* and *CDY* gene, and one primer pair for *SRY*. Primers were designed using Y chromosome sequences for *P. troglodytes* obtained from GenBank (NC_006492.2 – [4]) and Primer3 software. In order to obtain a more standardized amplification level, amplicon length was set at 110–150 bp. Homodimers and heterodimers, potential hairpins, and secondary structure formations were checked using Oligo Analyzer software; primer specificity was checked by UCSC In-Silico PCR on-line software. Primer sequences used for *DAZ*, *CDY* and *SRY* are summarized in Table S8. All qPCR reactions were carried out with 5 µl of QuantiTec SYBR^®^ Green PCR Master Mix (Qiagen) and 0.25 µM of each primer in a total volume of 10 µl. Assays included DNA standards at a final concentration of 5, 2.5, 1.25, 0.625 and 0.3125 ng/µl, a non-template control, and a female negative control. All assays were performed in duplicate. Cycling conditions were 50°C for 2 min, 95°C for 15 min, and 35 cycles of 95°C for 15 s, 55°C for 30 s and 72°C 30 s. Melting curve analysis was performed routinely following amplification to detect non-specific products. Each experiment was performed twice in independent runs to confirm the results. All qPCR reactions were carried out in an ABI Prism 7700 Sequence Detection System (Applied Biosystems). For each run, a standard curve was constructed by plotting the cycle number (Ct) versus the log of the DNA quantity. Genomic DNA from one of the bonobo specimens (Desmond) was used to construct the standard curve. The two-copy status of the *DAZ* gene in the bonobo Desmond was verified by Southern blotting [70] and FISH analysis [11]. The number of *DAZ* and *CDY* genes was calculated by interpolation of the Ct value in the corresponding standard curve using SDS 2.2.1 software (Applied Biosystems). In all cases, the number of copies of *DAZ* or *CDY* was normalized to *SRY* which was used as an internal control since it is known to be a single copy gene located on the Y chromosome. Mean values were calculated for each sample. Standard errors (SE) were calculated from the standard deviations (SD) of the normalized *DAZ* and *CDY* copy number values. Comparisons between the mean values were performed using the Student's unpaired *t*-test and a p-value <0.05 was considered significant.

## Supporting Information

Table S1 Gorilla specimens.
(DOC)

Table S2 Bornean orangutan (*Pongo pygmaeus*) specimens.
(DOC)

Table S3 Sumatran orangutan (*Pongo abelii*) specimens.
(DOC)

Table S4 Common chimpanzee – qPCR calculated data.
(XLS)

Table S5 Bonobo – qPCR calculated data.
(XLS)

Table S6 Bonobo – qPCR and FISH for *DAZ* & *CDY.*
(DOC)

Table S7 Common chimpanzee – qPCR and FISH for *DAZ* & *CDY.*
(DOC)

Table S8 Primer sequences.
(XLS)
