# Supplementary material for: Y-Chromosome Variation in Hominids: Intraspecific Variation Is Limited to the Polygamous Chimpanzee
Source: PLoS One. 2011 Dec 27;6(12):e29311. doi: 10.1371/journal.pone.0029311 (PMC3246485; doi:10.1371/journal.pone.0029311)
Supplement: Table S1 — Gorilla specimens. (DOC) [file pone.0029311.s001.doc]

**Table S1: Gorilla specimens**

| **Name** | **Stud #** | **Zoo** | **Birth Date** | **Notes** |
| --- | --- | --- | --- | --- |
| Awali | 1279 | Amsterdam | 24.04.1993 | Son of Tembo #185; wb ~1961 |
| Awembe | 1555 | Hannover | 08.11.2000 | Grandson of Ernst #566; wb ~1972 |
| Banjo | 255 | Stuttgart | wb ~1965 |  |
| Djengi | 1568 | Bristol | 26.06.2000 | Grandson of Matze #337; wb ~1957 |
| Efata | 793 | Stuttgart | 17.04.1982 | Son of Banjo #255; wb |
| Fritz | 253 | Nürnberg | wb ~ 1963 |  |
| Gincko | 1658 | Stuttgart | 17.07.2001 | Grandson of Bitam #458; wb ~1971 |
| Gaidi | 475 | Leipzig | wb ~1970 |  |
| Ivo | 1060 | Munich | 29.01.1988 | Son of Roututu #519; wb ~1973 |
| Kaisi * | 9909 | Antwerp | wb ~1955 |  |
| Kiondo | 1706 | Barcelona | 11.10.2002 | Grandson of Ernst #566; wb ~1972 |
| Kivu | 1707 | Barcelona | 27.11.2002 | Grandson of Ernst #566; wb ~1972 |
| Kukuma | 1089 | Apeldoorn | 29.09.1989 | Grandson of Colonel #184; wb ~1961 |
| Kumbuka | 1447 | Belfast | 15.11.1997 | Son of Keke #857; wb ~1981 |
| Noel | 942 | La Palmyr | 28.12.1986 | Grandson of Christophe #38; wb ~1949 |
| PolePole | 1106 | Zürich | 28.12.1989 | Grandson of Christophe #38; wb ~1949 |
| Rafiki | 1177 | Zürich | 29.05.1991 | Grandson of Christophe #38; wb ~1949 |
| Schorsch | 435 | Nürnberg | 03.03.1972 | Son of Fritz #253; wb |
| Simsim | 910 | Yerkes | 25.01.1986 | Son of Rann #232; wb ~1963 |
| Willy | 621 | Nürnberg | 21.07.1976 | Son of Fritz #253; wb |
| Yangu | 824 | Stuttgart | 24.05.1983 | Son of Banjo #255; wb |

International Gorilla Studbook, Mainlist, 31 December 2007

***** *Gorilla beringei graueri*; all other specimens: *Gorilla gorilla gorilla*

wb: wild-born
