# Supplementary material for: Y-Chromosome Variation in Hominids: Intraspecific Variation Is Limited to the Polygamous Chimpanzee
Source: PLoS One. 2011 Dec 27;6(12):e29311. doi: 10.1371/journal.pone.0029311 (PMC3246485; doi:10.1371/journal.pone.0029311)
Supplement: Table S2 — Bornean orangutan (Pongo pygmaeus) specimens. (DOC) [file pone.0029311.s002.doc]

**Table S2: Bornean orangutan (*Pongo pygmaeus*) specimens**

| **Name** | **Stud #** | **Zoo** | **Birth Date** | **Notes** |
| --- | --- | --- | --- | --- |
| Benjamin | 1601 | Wuppertal | 06.11.1977 | Son of Max #793; wb ~1960 |
| Jantan | 1700 | Krefeld | 03.03.1979 | Son of Jod #564; wb ~1961 |
| Jonny | 1236 | Cologne | 21.05.1972 | Son of Eddi #358; wb ~1958 |
| Kajan | 1610 | Berlin | wb ~1969 |  |
| Kenallen | 171019 | SanDiego | 06.11.1977 | Son of Bob #158144; wb ~1955 |
| Bulu* | 1741 | Basel | 29.12.1979 | Son of Niko #153; wb ~1950 |
| Maias | 1052 | Cologne | wb ~1967 |  |
| Napoleon | 898 | Studen CH | wb ~1965 |  |
| Sandai | 2617 | Cologne | 20.08.1993 | Grandson of Maias #1052; wb |
| Thai | 2861 | Duisburg | 25.04.1998 | Grandson of Pi-Ku #379; wb ~1958 |
| Tom | 2256 | Studen CH | 13.03.1989 | Son of Napoleon #898; wb |
| Vandu | 2672 | Stuttgart | 23.11.1994 | Grandson of Maias #1052; wb |
| Yogi/Joki | 825 | Cologne | wb ~1964 |  |

2002 International studbook of the orang-utan (Pongo pygmaeus, Pongo abelii); Lori Perkins, studbook keeper, Lincoln Park Zoo, 2001 North Clark Street, Chicago, IL 60614, USA.

Orang-Utan Europäisches Erhaltungszuchtprogramm, Zuchtbuch für Europa XXVI/2008; Clemens Becker, Zuchtbuchführer und EEP-Koordinator, Zoo Karlsruhe, Ettlinger Strasse 6, D-76137 Karlsruhe.

wb: wild-born

* hybrid *Pongo pygmaeus* (father) and *Pongo abelii* (mother)
