# Supplementary material for: Y-Chromosome Variation in Hominids: Intraspecific Variation Is Limited to the Polygamous Chimpanzee
Source: PLoS One. 2011 Dec 27;6(12):e29311. doi: 10.1371/journal.pone.0029311 (PMC3246485; doi:10.1371/journal.pone.0029311)
Supplement: Table S3 — Sumatran orangutan (Pongo abelii) specimens. (DOC) [file pone.0029311.s003.doc]

**Table S3: Sumatran orangutan (*Pongo abelii*) specimens**

| **Name** | **Stud #** | **Zoo** | **Birth Date** | **Notes** |
| --- | --- | --- | --- | --- |
| Bassir II*^,2^ | 2423 | Studen CH | 21.09.1991 | Grandson of Bassir #587; wb ~1960 |
| Benjamin^2^ | 1516 | Stuttgart | 26.05.1976 | Son of Buschi #498; wb |
| Bruno^1^ | 1000 | Munich | 18.02.1969 | Son of Max #231; wb ~1954 |
| Buschi^2^ | 498 | Stuttgart | wb ~1959 |  |
| Charly^1^ | 497 | Frankfurt | wb ~1957 |  |
| Duapuluh^1^ | 2810 | Munich | 06.10.1997 | Son of Bruno #1000 |
| Hummel^1^ | 411 | Nürnberg | wb ~1957 |  |
| Jolo^1^ | 1254 | Duisburg | 02.10.1972 | Son of Major #1016; wb ~1962 |
| Masala^2^ | 2404 | Stuttgart | 21.01.1991 | Son of Benjamin #1516 |
| Moritz^1^ | 2522 | Stuttgart | 20.12.1992 | Son of Bruno #1000 |
| Pandai^1^ | 3368 | Frankfurt | 13.09.2009 | Son of Charly #497; wb |
| Pendek*^,2^ | 1636 | Berlin | 23.01.1978 | Grandson of Tuan #538; wb ~1950 |
| Schubbi^1^ | 1237 | Basel | 28.05.1972 | Son of Lipis #534; wb ~1957 |
| Sinjo^2^ | 1007 | Hamburg | 16.03.1969 | Son of Tuan #538; wb ~1950 |
| Sumbo*^,1^ | 1942 | Berlin | 13.10.1983 | Son of Tarzan #127; wb ~1950 |
| Tuan/Tao^1^ | 2865 | Duisburg | 23.05.1998 | Son of Siam #543; wb ~1960 |
| Ujian^1^ | 2664 | Stuttgart | 25.06.1994 | Son of Pongo #787; wb ~1961 |

2002 International studbook of the orang-utan (Pongo pygmaeus, Pongo abelii); Lori Perkins, studbook keeper, Lincoln Park Zoo, 2001 North Clark Street, Chicago, IL 60614, USA.

Orang-Utan Europäisches Erhaltungszuchtprogramm, Zuchtbuch für Europa XXVII/2009; Clemens Becker, Zuchtbuchführer und EEP-Koordinator, Zoo Karlsruhe, Ettlinger Strasse 6, D-76137 Karlsruhe.

wb: wild-born

* hybrid *Pongo pygmaeus* (mother) and *Pongo abelii* (father)

^1^ Yqs-type 1: satellited Y with FISH-signals for *DAZ* and *CDY* in Yq only

^2^ Yqs-type 2: satellited Y with FISH-signals for *DAZ* and *CDY* in Yq and Yp
