# Supplementary material for: Y-Chromosome Variation in Hominids: Intraspecific Variation Is Limited to the Polygamous Chimpanzee
Source: PLoS One. 2011 Dec 27;6(12):e29311. doi: 10.1371/journal.pone.0029311 (PMC3246485; doi:10.1371/journal.pone.0029311)
Supplement: Table S6 — Bonobo – qPCR and FISH for DAZ & CDY. (DOC) [file pone.0029311.s006.doc]

**Table S6: Bonobo – qPCR and FISH for *DAZ* & *CDY***

| **Name** | **Stud #** | **qPCR / copies**  *DAZ* *CDY* | | **FISH / signals ***  *DAZ**CDY* | |
| --- | --- | --- | --- | --- | --- |
| Masikini | 54 | 2 | 2 | 1 | 1 |
| Limbuko | 210 | 2 | 2 | 1 | 1 |
| Kirembo | 177 | 2 | 2 | 1 | 1 |
| David | 277 | 2 | 2 | 1 | 1 |
| Mofana | 84 | 2 | 2 | 1 | 1 |
| Jasongo | 159 | 2 | 2 | 1 | 1 |
| Desmond | 57 | 2 | 2 | 1 | 1 |
| Yenge | 111 | 2 | 2 | 1 | 1 |
| Zorba | 168 | 2 | 2 | 1 | 1 |
| Bono | 102 | 2 | 2 | 1 | 1 |

* data from Schaller et al. 2010
