# Supplementary material for: Y-Chromosome Variation in Hominids: Intraspecific Variation Is Limited to the Polygamous Chimpanzee
Source: PLoS One. 2011 Dec 27;6(12):e29311. doi: 10.1371/journal.pone.0029311 (PMC3246485; doi:10.1371/journal.pone.0029311)
Supplement: Table S7 — Common chimpanzee – qPCR and FISH for DAZ & CDY. (DOC) [file pone.0029311.s007.doc]

**Table S7: Common chimpanzee – qPCR and FISH for *DAZ* & *CDY***

| **Name** | **Stud #** | **qPCR / copies**  *DAZ* *CDY* | | **FISH / signals ***  *DAZ**CDY* | |
| --- | --- | --- | --- | --- | --- |
| Bimbo |  | 2 | 6 | 1 | 4 |
| Tommy |  | 2 | 2 | 1 | 2 |
| Tim |  | 2 | 2 | 1 | 2 |
| Bobby |  | 2 | 3 | 1 | 2 |
| Sascha | 12527 | 2 | 2 | 1 | 2 |
| Moritz | 10920 | 2 | 2 | 1 | 2 |
| Max(1) |  | 2 | 3 | 1 | 2 |
| Mike |  | 2 | 2 | 1 | 2 |
| Max(2) | 12914 | 4 | 4 | 2 | 3 |
| Fritz |  | 2 | 3 | 1 | 3 |

* data from Schaller et al. 2010

Note: Members of the Y-specific ampliconic DAZ gene family are always arranged as inverted tandem duplications connected with a non-duplicated short spacer within palindromes (Skaletsky et al. *The male-specific region of the human Y chromosome is a mosaic of discrete sequence classes*. Nature, 423:825-837, 2003; Hughes et al. *Chimpanzee and human Y chromosomes are remarkably divergent in structure and gene content*. Nature, 437:100-103, 2010). These tandem duplications of DAZ are beyond the resolution of FISH, meaning that a single FISH signal corresponds to 2 DAZ genes and that DAZ copies always occur in even numbers.

In contrast, the members of the ampliconic CDY gene family either can be arranged in tandem, or as a single gene in chimpanzee (Hughes et al. *Chimpanzee and human Y chromosomes are remarkably divergent in structure and gene content*. Nature, 437:100-103, 2010). This explains, e.g. that 2 FISH-signals for CDY can correspond to 2 or to 3 CDY copies.
